# The Other Side of Chronic Venous Disorder: Gaining Insights from Patients’ Questions and Perspectives

**DOI:** 10.3390/jcm13092539

**Published:** 2024-04-26

**Authors:** Daniele Bissacco, Chiara Pisani

**Affiliations:** Department of Clinical Sciences and Community Health, University of Milan, 20122 Milan, Italy

**Keywords:** chronic venous disease, CEAP, great saphenous vein, varicose veins

## Abstract

Venous disorders encompass a diverse range of manifestations and diseases, impacting a significant portion of the population. While life-threatening conditions are uncommon in non-thrombotic disorders, like telangiectasias or uncomplicated varicose veins (VVs), these conditions still have a substantial impact on affected individuals. Ensuring that patients are well informed about their venous disorder is a crucial step in their treatment journey. Providing them with valuable information regarding the disease’s natural progression and available therapeutic options plays a pivotal role in optimizing their care. When patients are diagnosed with venous disorders, they often have numerous questions and concerns they want to discuss with their healthcare providers. Addressing these inquiries not only improves patients’ knowledge and understanding but also influences their treatment compliance and overall outcomes. Therefore, it is of utmost importance to provide comprehensive explanations that address any doubts, uncertainties, and areas of confusion that patients may have. This report aims to present a concise, practical, and informative guide to venous disorders, focusing specifically on the common questions frequently raised by patients in everyday clinical practice. By serving as a valuable resource for healthcare professionals working in the field of venous diseases, this guide equips them with the necessary tools to effectively address patients’ concerns and provide optimal care. By bridging the gap between patients’ inquiries and medical expertise, this guide strives to enhance therapeutic outcomes and improve the overall management of venous disorders, ultimately empowering patients in their treatment journey.

## 1. Introduction

Venous disorders encompass a wide spectrum of manifestations and diseases, and while life-threatening conditions are rare in non-thrombotic disorders, like telangiectasias or uncomplicated varicose veins (VVs), these conditions still affect a significant portion of the general population. When a venous disorder is diagnosed, one of the crucial steps is to ensure that patients are well informed about their condition. Providing them with valuable information and explaining the natural progression of the disease, as well as the available therapeutic options, plays a pivotal role in their treatment journey. In this context, patients often have numerous questions and concerns that they want to discuss with their healthcare providers. Addressing these queries can have a profound impact on patients’ knowledge and understanding, which in turn can influence their treatment compliance and overall outcomes. It is of utmost importance to provide comprehensive explanations that address any doubts, uncertainties, and areas of confusion that patients may have.

The primary objective of this report is to offer a concise, practical, and informative guide to venous disorders, starting from the common questions raised by patients in everyday clinical practice. This guide aims to serve as a valuable resource for healthcare professionals working in the field of venous diseases, equipping them with the necessary tools to effectively address patients’ concerns and provide optimal care. By bridging the gap between patients’ inquiries and medical expertise, this guide strives to enhance therapeutic outcomes and improve the overall management of venous disorders.

### 1.1. Doctor, How Are the Veins of the Lower Limbs Made and Which Ones Get Sick?

**Figure 1:**
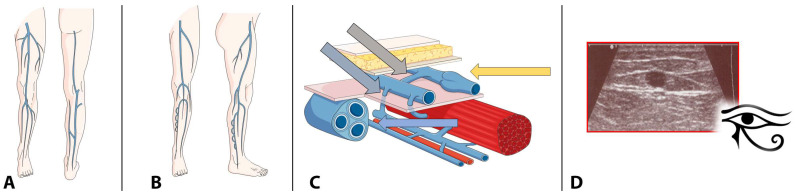
(**A**), superficial venous system; (**B**), deep venous system; (**C**), venous compartments: above the fascia (yellow arrow) and below the muscular fascia (blue arrow); connecting different or communicating the same compartments (grey arrows); (**D**), saphenous fascia, similar to the Eye of Horus.

**Figure 2:**
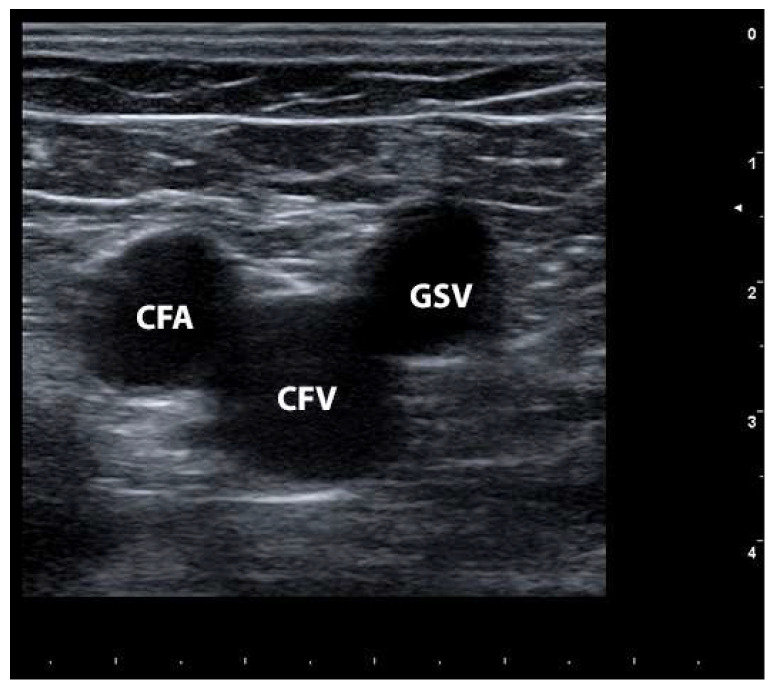
Mickey Mouse sign. CFA: common femoral artery; CFV: common femoral vein; GSV: great saphenous vein.

The circulatory system of the lower extremities comprises various venous components that play critical roles in blood drainage and return to the heart (Figure 1). The superficial venous system, as depicted in Figure 1A, primarily facilitates the drainage of blood from the skin and subcutaneous tissues. Traditionally, veins located above the deep muscular fascia, excluding deep veins (Figure 1B), are categorized as superficial veins. This system can be further divided into two main components: thick-walled truncal veins, such as the great saphenous vein (GSV) and the small saphenous vein (SSV), which lie between the saphenous sheath and the muscular fascia, and thin-walled superficial or epifascial tributaries, situated between the skin and saphenous fascia (Figure 1C). The relationship between these veins and their associated sheaths or fascia resembles the shape of the Eye of Horus when visualized using ultrasound (Figure 1D). This anatomical feature serves as a crucial marker for identifying the saphenous veins. However, it is important to note that only around 50% of patients exhibit complete distribution of the saphenous trunk throughout the entire saphenous compartment, spanning from the ankle to the groin [1]. The GSV, which is the longest vein in the body, originates on the medial side of the foot and ascends anteriorly to the medial malleolus, subsequently coursing along the medial side of the calf and thigh, and ultimately draining into the common femoral vein (CFV). In the tibial region, the GSV receives contributions from two tributaries: the posterior accessory of the GSV (the PASV, previously referred to as the vein of Leonardo) and the anterior accessory of the GSV (the AASV). The saphenofemoral junction (SFJ), also known as the saphenous junction or saphenous arch, represents a critical area in terms of understanding flow patterns, treatment approaches, and varicosity recurrence. Ultrasound imaging allows for the identification of the “Mickey Mouse” sign, as depicted in Figure 2, which serves as a landmark consisting of the common femoral artery, CFV, and the GSV.

**Figure 3:**
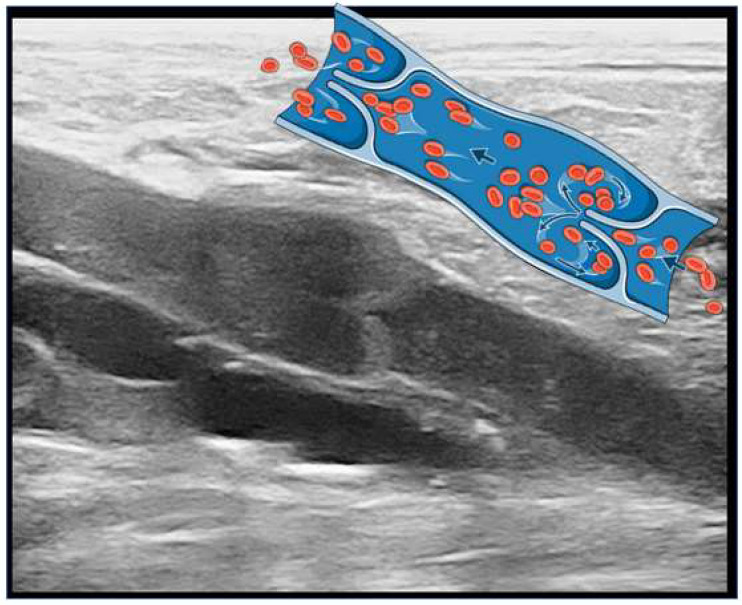
Venous valve located in the popliteal vein (arrows indicate venous blood flow direction).

The SFJ encompasses the region between the terminal and preterminal valves of the CFV, along with three major tributaries that drain into the GSV: the external pudendal, inferior epigastric, and external circumflex iliac veins. Notably, the SFJ exhibits significant anatomical variation [2]. The AASV serves as a major distal tributary of the SFJ. Approximately half of patients possess the AASV, which can be a source of recurrent VVs, making it a crucial consideration in diagnosis and treatment [3]. On the other hand, the SSV originates from the dorsolateral aspect of the foot and ascends posteriorly to the lateral malleolus, continuing along the posterolateral aspect of the calf, and eventually draining into the popliteal vein. The saphenopopliteal junction (SPJ), where the SSV joins the popliteal vein, exhibits variability [4]. Cranial extension of the SSV, which represents the continuation of the vein, is present in approximately 95% of limbs. However, in about 25% of limbs, the SSV lacks a connection to the deep vein and continues up the posterior thigh as the cranial extension. The SPJ is typically located above the popliteal crease but may be situated at a higher point in 25% of limbs. Moving on to the deep venous system (Figure 1B), it functions as a low-pressure, high-volume system responsible for approximately 90% of the venous blood flow in the lower extremities. Deep veins generally possess thinner walls compared to superficial veins but are supported by surrounding muscles and/or fascia, forming a rigid compartment that aids in propelling venous blood upward during walking. In general, deep veins follow the course of corresponding arteries, except in the distal region of intramuscular veins such as the soleal and gastrocnemius veins. The deep venous system in the infrapopliteal area consists of the anterior and posterior tibial veins, the peroneal vein, the soleal vein, and the gastrocnemius vein. The primary function of the deep venous system is to facilitate the return of deoxygenated blood from the lower extremities back to the heart. The deep veins work in conjunction with the superficial veins to ensure efficient blood circulation. The main deep veins in the lower extremities include the tibial, soleal (always in pairs), popliteal, superficial femoral/deep femoral, and common femoral veins. They have valves (Figure 3) that help maintain one-way blood flow, preventing backflow and facilitating venous return. Dysfunction or blockage of these deep veins can lead to conditions such as deep vein thrombosis (DVT), which is the formation of blood clots in the deep veins.

Perforating veins (PVs), also known as communicating veins, are a network of veins that connect the superficial veins to the deep veins. They play an important role in the circulation of blood and have one-way valves that prevent the backward flow of blood from the superficial veins to the deep veins, against gravity, and during muscle contraction and movement. However, if the valves become damaged or incompetent, blood can flow in the opposite direction, resulting in venous reflux and contributing to the development of venous insufficiency and VVs.

In most cases, if DVT is not present, venous disease involves one or more saphenous trunk (GSV, SSV, AASV, PASV) and/or their tributaries and/or PVs.

### 1.2. Doctor, What Is Chronic Venous Disease and What Are Varicose Veins?

**Figure 4:**
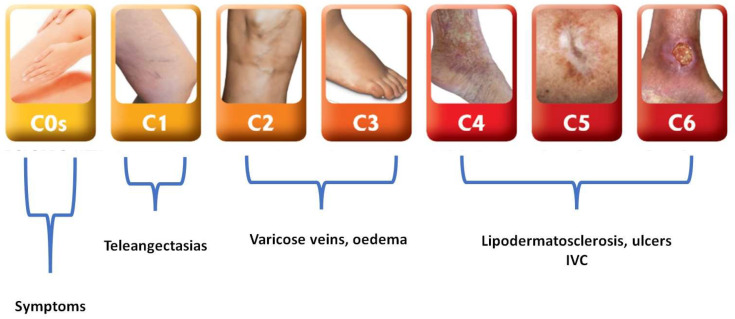
CEAP C classes. CVI: chronic venous insufficiency.

The term chronic venous disease (CVD) has been defined as “(any) morphological and functional abnormalities of the venous system of long duration manifest either by symptoms and/or signs indicating the need for investigation and/or care” [5]. Given that not all venous abnormalities can be categorized as a distinct “disease”, it has become necessary to introduce the term “chronic venous disorders” to encompass the entirety of morphological and functional abnormalities that can occur within the venous system. Patients who exhibit symptoms and/or signs indicative of chronic venous disorders necessitate further investigation and/or medical attention. To classify CVD, the CEAP (Clinical, Etiologic, Anatomic, and Pathophysiologic) classification should be used for describing signs and symptoms related to lower limb vein disorders [6,7]. In particular, the Clinical (C) classification (Figure 4) stages include the following:

C0s—symptoms of CVD with no visible or palpable signs on clinical examination: The description of and relationship between venous symptoms in the legs and CVD have sparked controversy. It has been observed that venous symptoms can occur across all classes of CVD (ranging from C0s—in which “s” indicates the presence of symptoms—to C6s), with various studies identifying a significant correlation between these symptoms and the worsening of CVD signs, ultimately impacting the quality of life (QoL) of individuals. However, findings from the Edinburgh Vein Study [8] have challenged this association by failing to demonstrate a link between “venous” leg symptoms and VVs, suggesting that most of these symptoms may have non-venous origins. Furthermore, there appears to be a weak correlation between symptoms and signs, as well as routine investigation results. The most common leg symptoms experienced in CVDs include aching, pain, heaviness, and discomfort [9]. Notably, the intensity of pain or ache does not necessarily align with the clinical severity of the condition, and the pain or ache is often described as dull, diffuse, and challenging to precisely pinpoint. Patients frequently encounter difficulty in describing the quality of the pain, often resorting to vague terms that are not typically used to characterize pain. Additionally, other symptoms, such as throbbing, tightness, fatigue, a sense of swelling, cramps, itching, restless legs, and tingling sensations, may also be present. Unfortunately, there exists a widespread lack of clarity regarding symptoms, coupled with the inappropriate use of terminology within the medical community, leading to frequent misunderstandings in the physician–patient relationship.

C1—telangiectasias or reticular veins: telangiectasias refer to the dilation of intradermal venules, characterized by their width measuring below 1 mm. On the other hand, reticular veins are dilated bluish subdermal veins, with a diameter ranging from 1 mm to 3 mm [6,7]. It is noteworthy that the prevalence of reticular veins and telangiectasias within the adult population is relatively high [10]. Although these vascular abnormalities have been linked to the existence of underlying significant venous system incompetence, it is important to acknowledge that their treatment primarily stems from aesthetic considerations [11].

C2—varicose veins: VVs are one of the most prevalent signs of CVD. They are enlarged, twisted, and swollen veins that usually occur in the legs and feet. VVs often appear as bulging, twisted cords beneath the skin. They may be blue, purple, or red in color and can be accompanied by symptoms such as aching, throbbing, or a heavy sensation in the affected area [12]. Other symptoms may include swelling, muscle cramps, and itching around the veins. They can be present according to CVD pattern. They are usually derived as tributaries of an incompetent saphenous vein (GSV, SSV, or both) or as collateral from a PV.

C3—edema: it is characterized by a noticeable increase in the volume of fluid in the skin and subcutaneous tissue, which can be visibly indented by applying pressure [13]. Despite this clear definition, the incidence of stage C3 also varies due to it having the lowest level of agreement found for class C3. Despite this, there are some specific findings.

C4—skin changes (e.g., eczema or lipodermatosclerosis).

C5—healed venous leg ulceration (VLU). 

C6—active VLU.

### 1.3. How Many People Suffer from CVD?

According to the available data deriving from a systematic review of published studies [14], the prevalence rates for each stage of C classification in chronic venous disease are as follows: C0s: 9%; C1: 26%; C2: 19%; C3: 8%; C4: 5%; C5: 1%; and C6: 0.42%. When considering the overall burden of C0s–C6 diseases, estimates range from 45.6% to 83.6%. However, if we exclude C0s, the estimates for C1–C6 range from 38.3% to 90.4% of the population based on various studies. The prevalence of C2 disease has been reported for different geographic regions. In Europe, the pooled prevalence is approximately 21%, while in Asia, it is approximately 17% based on studies conducted in the Middle East and one study that also included patients from Southeast Asia (Indonesia, Thailand, Singapore, Vietnam). The reported prevalence in Africa is 5.5%, in North America it is 23%, in South America it is 22%, and in the Pacific Islands it is 19%. Regarding the annual incidence of C2 disease, it ranges from 0.22% to 2.3%. For C5/C6 diseases, the annual incidence ranges from 0.018% to 0.122% in the general adult population and from 0.3% to 1.2% in older adults.

However, it must be considered that prevalence and incidence rates are based on relatively few available studies and may vary in different populations and regions.

### 1.4. How Is It Possible to Diagnose CVD?

The physical examination of a patient with lower extremity venous disease involves a general examination and a detailed examination of the legs. The examination begins with visual inspection and palpation of the legs. The location and distribution of VVs should be noted and recorded, including the main saphenous trunk and spider veins. Swelling (edema) and abnormal blood vessels (angiomatous malformations) should also be checked. Large VVs over sites of perforating veins should be identified. Palpation of the legs may reveal additional varicose veins that are not easily visible. The internal malleolar area remains one of the most important for the clinical evaluation of CVD, because it could be the location of healed or active ulcers, as well as skin discoloration and lipodermatosclerosis (CEAP C4–6). Several tests can be performed during the initial evaluation in an outpatient setting to provide clues about the underlying cause of venous hypertension, such as the cough impulse test; the tap test, also known as the percussion test; the Perthes test; and the Brodie–Trendelenburg test [15]. These clinical tests have been mostly replaced by more advanced diagnostic imaging tests like Color Doppler Ultrasound (CDUS) scans. A commonly performed test in the office setting is hand-held continuous-wave Doppler ultrasound examination of the GSV/SSV. This involves insonating the saphenous vein at the SFJ while an assistant applies compression to the calf. The presence of reflux upon release of the compression indicates incompetence of the target saphenous trunk (Figure 4). These diagnostic tests provide valuable information about venous incompetence and help guide further management. Other diagnostic tests (pletismography, venography, computed tomography, or magnetic resonance angiography) could be used for scientific purposes or in challenging cases, such as both superficial and deep CVD.

### 1.5. What Are the Treatments to Reduce CVD? Are They Always Invasive Treatments?

Varicose veins can be effectively treated using a wide range of medical procedures and therapies. Therapies for CVD can be divided into conservative treatment, which are usually proposed in patients with mild stages (CEAP C0–C1) or if contraindications to further treatments are present, and invasive treatments, which are used in addition to conservative ones, to achieve the best results in advanced stages (CEAP C2–6). Additionally, behavioral and lifestyle therapies, such as controlling weight and other vascular risk factors, physical activities with muscular pump stimulation, avoiding standing for long periods, etc., should be followed in all CEAP classes to minimize CVD progression and complications.

Compression therapy is a widely used treatment for varicose veins that aims to reduce pain and swelling. It involves the use of elastic compression stockings, which counteract tissue expansion during muscle contraction. These stockings work by narrowing the diameter of superficial veins, thereby alleviating venous reflux and venous hypertension, which are important factors in the development of CVD. Graduated compression stockings are available with different pressure levels, ranging from low-pressure Class 1 stockings to higher-pressure options (Classes 2, 3, and 4). According to recent American and European guidelines, compression therapy is recommended as the primary treatment for patients with symptomatic varicose veins and axial reflux in the superficial truncal veins, especially if the patient’s ambulatory status or underlying medical conditions warrant a conservative approach [5,16]. European guidelines additionally recommend elastic compression stockings for reducing venous symptoms, edema, and skin induration in patients with symptomatic chronic venous disease [5].

Venoactive drugs (VADs) encompass a range of medications that provide benefits for various CVD conditions. They primarily act on venous wall inflammations, which are key pathological mechanisms in the development of CVD [17]. Among the different classes of VADs, flavonoids such as rutin (Oxerutin) and micronized purified flavonoids fraction (MPFF) have solid scientific evidence supporting their effectiveness in relieving symptoms and signs associated with CVD [18,19].

Endovenous thermal techniques, including laser ablation (EVLA) and radiofrequency ablation (RFA), are considered the gold standard for treating incompetence of the GSV or SSV. These procedures involve using endovenous energy to heat the blood vessels, damaging the inner layers of the vein walls and blocking blood flow. EVLA and RFA are highly effective and safe, with fewer complications compared to traditional stripping and surgery [5,16]. They can be performed under local anesthesia and do not require a hospital stay. Care must be taken during the procedure to prevent skin burns and deep vein thrombosis by maintaining distance between the vein and the skin [20].

Foam sclerotherapy is another technique used to treat various venous diseases, including telangiectasias, varicose veins, and saphenous trunks. It involves injecting a foam sclerosant into the affected vein, causing an inflammatory reaction, cell death, endothelial damage, and vein closure [21]. While foam sclerotherapy is effective for the aesthetic removal of telangiectasias and is cost-effective, its outcomes are generally lower compared to endovenous thermal techniques [5,16].

Surgery, specifically saphenous trunk stripping and high ligation (S + HL), is recommended only when other techniques are contraindicated. It involves the removal of the GSV or SSV through incisions made in the groin, popliteal area, and leg. Phlebectomy is another surgical technique used to remove varicose veins through small incisions made along the affected veins. The procedure uses a phlebectomy hook and forceps to trap and remove the varicose veins after ligation.

**Figure 5:**
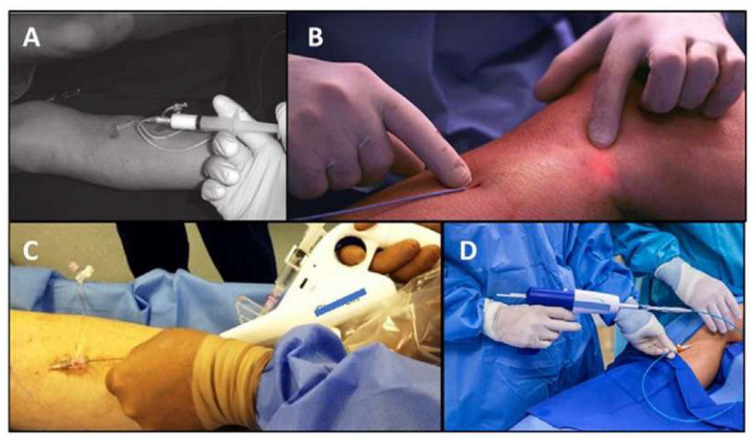
Techniques for varicose vein endovenous ablation. (**A**) Sclerotherapy. (**B**) Endovenous laser ablation. (**C**) Mechanochemical ablation. (**D**) Cyanoacrylate embolization.

Other techniques, such as glue injection (N-butyl-cyanoacrylate), mechanochemical ablation, or CHIVA (Conservatrice Hémodynamique de l‘Insuffisance Veineuse en Ambulatoire), have been suggested [22] but have not shown comparable results to EVLA/RFA in the scientific literature. Figure 5 shows some of techniques used to treat saphenous trunk insufficiency.

### 1.6. What Are the Risk Factors That Contribute to CVD?

CVD can be classified into two main categories: primary and secondary. Primary CVD refers to cases where there is no identifiable triggering event or cause for the disease. On the other hand, secondary CVD occurs because of a specific event or induction. In both onsets, it is influenced by a variety of predisposing factors, including both genetic and environmental elements. Numerous studies have demonstrated the correlation between age and the prevalence and progression of primary CVD, particularly in older populations, where the occurrence ranges from 0.45% to 1.69% [23]. Furthermore, the onset of CVD has been found to be significantly associated with gender, with females experiencing initial symptoms at an average age of 30.8 years and males at 36.8 years [14]. Various hormonal and physiological factors contribute to the heightened susceptibility of women to develop CVD, with pregnancy being a significant contributor [24]. During pregnancy, a range of hemodynamic changes occur, such as expanded blood volume, reduced blood velocity, compression of the iliac veins due to the enlarging uterus, stasis, alterations in hormonal levels, smooth muscle relaxation, and vasodilatation. These changes collectively contribute to the development of varicose veins, and approximately 40% of pregnant women suffer from CVD [25]. The risk of developing varicose veins is also influenced by the number of pregnancies, with an estimated 20% of nulliparous women over 40 years old experiencing varicose veins. However, this percentage increases to 40% for women who have been pregnant one to four times and up to 65% for those with five or more pregnancies. A significant risk factor for CVD is a positive family history, indicating a high heritability of the disease and suggesting a notable genetic component in its etiology. However, the specific genes involved in CVD have not yet been identified [26]. It is important to note, though, that while these risk factors may contribute to the formation of varicose veins, not all individuals exposed to these factors will develop the disease. This suggests that environmental exposure and various habits can strongly influence the onset of CVD. Factors such as diet, obesity, lack of regular exercise, prolonged sitting or standing, and smoking are among the environmental predisposing elements [27]. Obesity is directly linked to the onset of CVD due to several synergistic effects, including the pro-inflammatory state associated with increased adiposity and factors like elevated intra-abdominal pressure, which can lead to reflux, increased vein diameter, and venous pressure [28]. Additionally, certain positional habits can significantly influence the onset of the disease, as even 30 min of quiet standing is sufficient to trigger an inflammatory response in the venous wall. Prolonged standing at work, where venous pressure is higher compared to sitting and walking, can result in persistent venous stasis, increasing the risk of venous reflux and varicose veins. Furthermore, ligamentous laxity caused by conditions like flat feet or previous hernia surgery has shown the highest correlation, albeit nonmodifiable, with moderate or severe venous disease [26]. Regular physical activity has been shown to have a protective effect against CVD by improving blood circulation and strengthening the muscle pump [29]. Conversely, a Western diet lacking in fiber can induce constipation and increase intra-abdominal pressure, which can lead to dilation in both the superficial and deep venous systems over time [26]. Traditional cardiovascular risk factors, however, have shown little to no correlation with CVD [30].

Secondary CVD refers to conditions that result from previous events that cause damage to the veins. The most common cause of secondary CVD is a prior episode of DVT or superficial venous thrombosis (SVT), which can lead to the development of “post-thrombotic syndrome” (PTS), observed in 20–50% of patients following deep vein thrombosis [26]. Additionally, post-traumatic CVD, resulting from blunt or penetrating trauma, can affect the upper or lower limbs and should be acknowledged as a secondary cause of the disease.

Congenital venous pathology primarily arises from malformations and is often recognized as clinical syndromes such as Klippel–Trenaunay or Parkes-Weber syndrome. These conditions present a more complex clinical picture and require specialized management of the disease. Klippel–Trenaunay syndrome is a rare congenital disorder characterized by a triad of symptoms, including capillary malformations (port-wine stains), venous malformations, and soft tissue and bone overgrowth. The most common feature of Klippel–Trenaunay syndrome is the presence of port-wine stains, which are reddish or purplish birthmarks that usually affect one or more limbs [31]. These skin discolorations are caused by the abnormal dilation of small blood vessels near the surface of the skin. Venous malformations, which are abnormalities in the veins, can also be present and may lead to chronic venous insufficiency and varicose veins. In addition to vascular abnormalities, individuals with Klippel–Trenaunay syndrome may experience overgrowth of soft tissues and bones in the affected limb(s). This can lead to limb length discrepancies, muscle weakness, joint problems, and an increased risk of fractures.

Parkes-Weber syndrome is characterized by the presence of arteriovenous malformations (AVMs) that cause abnormal connections between arteries and veins. These AVMs result in excessive blood flow to the affected area, leading to the enlargement and engorgement of blood vessels. The condition primarily affects the limbs but can also involve other parts of the body, such as the face, trunk, or organs. The most common symptom of Parkes-Weber syndrome is a visible vascular malformation, which appears as a reddish or purple birthmark known as a port-wine stain. These birthmarks are caused by the dilation of small blood vessels near the surface of the skin. Unlike in other vascular malformation disorders, such as Klippel–Trenaunay syndrome, Parkes-Weber syndrome is characterized by the presence of high-flow AVMs, which can cause the affected limb to be warm, swollen, and have an increased pulse. In addition to visible vascular malformations, individuals with Parkes-Weber syndrome may experience other complications. These can include overgrowth of the affected limb, skeletal abnormalities, muscle weakness, pain, and an increased risk of developing blood clots or heart failure in severe cases [32]. It is important for individuals with congenital venous malformations to receive ongoing medical monitoring and coordinated care to address their specific needs and optimize their quality of life.

### 1.7. Doctor, Could You Please Provide Me with a List of the Most Common Symptoms and Signs Associated with CVD?

The symptoms of CVD can vary depending on the severity and stage. In the later stages of CVD, which are classified according to the CEAP classification system, symptoms tend to be more pronounced. In a study called the Bonn vein study, which involved 2624 individuals with CVD, it was found that 56.7% of the participants reported experiencing at least one symptom related to CVD. These symptoms were more commonly reported by women and increased in frequency with age [33].

It is important to note that the symptoms of CVD can be nonspecific, particularly in the early stages of the disease, and may not be solely attributed to venous issues. In fact, studies have shown that similar symptoms can be observed in non-venous conditions such as knee and hip arthrosis, peripheral arterial disease, and spinal disc herniation. This suggests that lower extremity symptoms can be nonspecific in nature [34].

However, there are specific manifestations that are more representative of chronic venous disease. These include reticular veins, telangiectasias (spider veins), and VVs [11]. Other common signs and symptoms of CVD include aching and throbbing sensations, leg tiredness or fatigue, itching, tingling, or burning sensations, and nocturnal leg cramps or restlessness. These symptoms are caused by chronic venous dysfunction and venous hypertension. Many patients find relief by elevating their legs, and symptoms can worsen during the summer months and women’s menstrual cycles.

In the later and more advanced stages of CVD, the inflammatory environment affects the skin and subcutaneous tissues, leading to more severe symptoms. These can include soft tissue edema, dermatitis, hyperpigmentation, lipodermatosclerosis (localized fibrosis and scarring), ulceration, skin erosion, and, potentially, hemorrhage, especially in cases of trauma. Trophic skin changes, such as thickened skin, hemosiderin deposition, atrophie blanche (white atrophic skin with surrounding capillaries), and ulceration or ulcer scars, are all manifestations of severe chronic venous insufficiency [35]. Lipodermatosclerosis and atrophie blanche may be considered pre-ulcerative stages. The presence of visible abnormal cutaneous blood vessels at the ankle, known as corona phlebectatica, is also a sign of advanced CVD.

As the disease progresses, symptoms tend to worsen, particularly in situations where venous pressure is increased, and lymphatic drainage is impaired. Venous ulceration can become complicated by infection, requiring long periods of advanced dressing for healing, often taking more than 9 months. This can significantly impact the patient’s daily activities, both physically and psychologically, as well as having socioeconomic implications [36]. Furthermore, skin changes associated with CVD can rarely lead to malignant degeneration, which is strongly associated with a worsening of quality of life [37]. Additionally, CVD increases the risk of developing SVT, which is significantly associated with an increased risk of DVT, particularly in the femoropopliteal segment. There is also a risk of pulmonary thromboembolism, which is a potentially life-threatening condition.

### 1.8. What Is the Best Treatment Option for CVD?

For symptom management and disease progression control, a comprehensive approach encompassing medical, physical, and surgical strategies should be contemplated. For example, compression therapy by graduated ECS exerting an ankle pressure ranging from 15 to 32 mmHg has proven effective in relieving symptoms in patients with C1s and C3s CEAP clinical classes by decreasing pain, heaviness, cramps, and edema related to CVD [38].

Physical exercise, aimed at enhancing lower limb muscle strength and ankle mobility, coupled with physiotherapy, can enhance overall mobility, facilitate weight loss, bolster the calf muscle pump, and expand the range of ankle movements, promoting venous return. Consequently, they have the potential to diminish leg edema, mitigate or prevent skin alterations induced by CVD, and alleviate symptoms and manifestations of post-thrombotic syndrome [5,16].

VADs remain one of the most effective solutions in reducing CVD symptoms, starting from the C0s patient class [18] to more advanced disease (C4–C6) [39]. Despite the guidelines being ill-defined and the vague level of evidence in VAD recommendations [40], some products have proved to be effective. Hydroxyethylrutoside (HR), also known as Oxerutin, a flavonoid compound, possesses antioxidant and anti-inflammatory properties through the inhibition of several signaling pathways, targeting the endothelial surface, walls, and endothelial permeability [41]. In particular, HR has demonstrated its effectiveness in reducing various common signs of CVD, including edema, ankle circumference, leg volume, and the number of healed ulcers [42]. Moving on to the symptoms of CVD, HR significantly reduces pain, cramps, restless legs, and heavy legs [42]. HR and MPFF are the most discussed and effective VADs in the literature, despite HR significantly improving venous microangiopathy, signs/symptoms, and edema when compared with MPFF [43,44].

Utilizing topical treatments may offer a valuable additional approach to alleviate the signs and symptoms associated with CVD. Glycosaminoglycan polysulfate offers a multitude of advantageous effects, especially in the context of thrombotic disease linked to VVs. It exhibits local antithrombotic properties, diminishes inflammation, facilitates the restoration of connective tissue, and hinders the formation of fresh superficial blood clots, while aiding in their absorption. It is crucial to emphasize that the utilization of glycosaminoglycan polysulfate should be complemented with adjuvant therapies, such as the concurrent use of compression stockings and low-molecular-weight heparin (LMWH) [45].

Various surgical strategies are indeed available to address the pathology, particularly when conservative management proves ineffective. These strategies encompass a range of therapeutic options tailored to the localization and extent of the disease. The most practiced interventional approach in these patients is endovenous thermal ablation (RFA vs. EVLA), which is used for saphenous vein incompetence; it is less invasive compared to traditional open surgery, with a lower rate of potential complications, such as hematomas, infections of the surgical wound, or saphenous nerve injury. Because of that, nowadays ultrasound-guided endovenous ablations are considered the gold standard, replacing all the previous techniques used in this field. In cases of patients with incompetent small or great saphenous veins, endovenous thermal ablation (EVTA) emerges as the preferred treatment modality [5,16]. However, non-thermal and non-tumescent techniques, such as ultrasound-guided foam sclerotherapy (UGFS), could serve as viable alternatives. In situations where these methods are not feasible, traditional open surgery for SSV remains a viable option.

Tributary intervention is commonly conducted alongside truncal ablation, particularly for large incompetent tributaries (>5 mm diameter), or independently when the competence of the truncal vein is confirmed via duplex ultrasonography. Moreover, it may be incorporated into specific treatment strategies aimed at preserving the saphenous trunk. When performing saphenous ablation, the potential requirement for additional adjunctive procedures should always be openly discussed with the patient to facilitate shared decision-making.

To treat peripheral VVs and tributaries, different options are available, often associated with EVTA. The most applied techniques are traditional phlebectomies, with multiple small longitudinal skin incisions performed under local anesthesia with a very low rate of complications [46], or the less invasive sclerotherapy, which is often well tolerated without the need for anesthesia in the ambulatory setting. The decision to choose ambulatory phlebectomy or ultrasound-guided foam sclerotherapy for varicose tributaries is largely influenced by the physician’s experience and preferences, as well as the expectations of the patient. According to a global survey on management strategies for patients with varicose veins (VVs), involving 211 physicians from 36 countries, phlebectomies were utilized as frequently as UGFS for treating refluxing tributaries. However, there was a tendency to prefer phlebectomies in cases where tributaries exhibited a large diameter, superficial course, or were visibly prominent, while UGFS was preferred in other scenarios (*p* < 0.001). In patients with skin changes, particularly lipodermatosclerosis (CEAP clinical class C4b), concomitant phlebectomies might be associated with delayed wound healing. In such instances, UGFS stands as a viable alternative option [47].

### 1.9. Doctor, What Are the Chances of the Disease Recurring after Treatment?

The recurrence risk of venous disease following treatment can fluctuate based on factors such as the initial disease severity, treatment method chosen, patient characteristics, and adherence to post-treatment care. There are multiple criteria to assess the success of varicose vein treatment. Patients experiencing partial or complete recanalization of the great saphenous vein post initial treatment might remain asymptomatic and may not require further intervention. However, in different studies, it appears that a significant proportion did require retreatment.

Several reviews examining treatments for varicose veins have brought attention to the persistent issue of a high recurrence rate in the treated limb over the long term [48]. This recurrence is often attributed to technical or strategic errors, natural disease progression, and other factors. Additionally, these reviews stress the importance of reassessing costs and ensuring proper indications for treatment. Moreover, the disparity in saphenous occlusion rates between different procedures, often used as a surrogate outcome, consistently remains below 10% in the mid-term. This minimal variation raises questions regarding the value and necessity of innovative and more costly treatments, especially when the results achieved are comparable to those of older and less expensive procedures [49].

When assessing clinical recurrence, defined as the reappearance of varicose veins in the treated limb, a recurrence rate of approximately 30–35% is still observed in the mid-term. Moreover, the CLASS trial has reported even higher rates of new varicose veins in the operated limbs at 5 years follow-up, ranging from 42% to 53% [50]. In this randomized, controlled trial conducted across 11 centers in the United Kingdom, involving 798 participants with primary varicose veins, the outcomes of laser ablation, foam sclerotherapy, and surgery were compared. By the 5-year mark, 58% of patients who underwent laser ablation, 54% who underwent surgery, and 47% who underwent foam sclerotherapy reported being free of varicose veins. Additionally, 11% of patients in the laser ablation group, 14% in the foam sclerotherapy group, and 7% in the surgery group required further treatment. This finding aligns with the results reported in other studies, like this RCT carried out at two university hospitals in Finland during 2008–2010, reporting 5-year follow-up results comparing open surgery, EVLA, and UGFS in the treatment of GSV reflux [51]. EVLA stands out as a safe and effective method for treating GSV reflux, with durable results. It exhibits a slightly lower rate of requiring additional treatments compared to surgery. On the other hand, UGFS resulted in anatomical success without further GSV treatment in only 27% of patients at 5 years (16 out of 59); patients in the UGFS group required more repeat treatments. Primary GSV reflux is best managed with either open surgery or an endothermal ablation method such as EVLA. While UGFS remains a valid option for those with recurrent varicose veins or incompetent tributary veins, its efficacy in treating primary GSV reflux is limited.

The limited literature available on long-term (10 years) outcomes indicates that surgery and/or sclerotherapy account for 37–56% of new varices, while stripping procedures result in a 66.5% clinical and/or duplex-based recurrence rate [52].

Limited data in the literature are available on extended follow-up (10 years or more) for new endovascular techniques, and further and larger studies are needed to establish their actual effectiveness in terms of recurrence and the need for reintervention.

## 2. Conclusions

Healthcare professionals can take several steps to effectively address patients’ concerns and provide optimal care for CVD:

Active Listening: Healthcare professionals should actively listen to patients and create a supportive and empathetic environment. Encourage patients to share their experiences, fears, and questions openly. This helps in understanding their concerns and tailoring the information and care accordingly.

Patient Education: Provide comprehensive patient education regarding the nature of venous disorders, their causes, progression, and available treatment options. Use clear and understandable language, visual aids, and other educational resources to facilitate understanding. Empower patients with knowledge about self-care measures, lifestyle modifications, and preventive strategies to manage their condition effectively.

Individualized Treatment Plans: Develop individualized treatment plans based on patients’ specific needs, symptoms, and preferences. Discuss the available treatment options and their benefits, risks, and expected outcomes. Involve patients in shared decision-making to ensure their active participation and collaboration in the treatment process.

Addressing Patient Concerns: Take the time to address patient concerns thoroughly. Answer questions, alleviate fears, and provide realistic expectations about the course of treatment and potential outcomes. Be patient and compassionate, offering reassurance and support throughout the process.

Clear Communication: Use clear and concise language when explaining medical concepts, procedures, and treatment plans. Avoid jargon or technical terms that may confuse patients. Encourage patients to ask questions and clarify any uncertainties they may have.

Collaborative Care: Work collaboratively with other healthcare professionals, such as vascular surgeons, nurses, and specialists, to ensure a multidisciplinary approach to care. This allows for the comprehensive evaluation and management of venous disorders, optimizing treatment outcomes.

Continuity of Care: Establish a long-term relationship with patients to provide ongoing support and monitoring. Schedule regular follow-up appointments to assess treatment progress, address any new concerns, and make necessary adjustments to the treatment plan.

Supportive Resources: Provide patients with additional resources, such as written materials, online sources, or support groups, where they can find further information and connect with others facing similar challenges.

Empathy and Emotional Support: Recognize the emotional impact of venous disorders on patients’ lives. Offer emotional support, understanding, and empathy. Acknowledge the physical and psychological burden of the condition and provide appropriate referrals for counseling or psychological support if needed.

By implementing these strategies, healthcare professionals can effectively address patients’ concerns, enhance their understanding of venous disorders, and provide optimal care that is tailored to their individual needs, ultimately improving treatment outcomes and overall patient satisfaction.

## Data Availability

Not applicable.
